# Hall effect in gated single-wall carbon nanotube films

**DOI:** 10.1038/s41598-021-03911-7

**Published:** 2022-01-07

**Authors:** Yohei Yomogida, Kanako Horiuchi, Ryotaro Okada, Hideki Kawai, Yota Ichinose, Hiroyuki Nishidome, Kan Ueji, Natsumi Komatsu, Weilu Gao, Junichiro Kono, Kazuhiro Yanagi

**Affiliations:** 1 Department of Physics, Tokyo Metropolitan University, Hachioji, Tokyo 192-0397 Japan; 2 Department of Electrical and Computer Engineering, Rice University, Houston, TX 77005 USA; 3 Department of Electrical and Computer Engineering, University of Utah, Salt Lake City, UT 84112 USA; 4 Department of Physics and Astronomy, Rice University, Houston, TX 77005 USA; 5 Department of Materials Science and NanoEngineering, Rice University, Houston, TX 77005 USA

**Keywords:** Carbon nanotubes and fullerenes, Electronic devices

## Abstract

The presence of hopping carriers and grain boundaries can sometimes lead to anomalous carrier types and density overestimation in Hall-effect measurements. Previous Hall-effect studies on carbon nanotube films reported unreasonably large carrier densities without independent assessments of the carrier types and densities. Here, we have systematically investigated the validity of Hall-effect results for a series of metallic, semiconducting, and metal–semiconductor-mixed single-wall carbon nanotube films. With carrier densities controlled through applied gate voltages, we were able to observe the Hall effect both in the *n*- and *p*-type regions, detecting opposite signs in the Hall coefficient. By comparing the obtained carrier types and densities against values derived from simultaneous field-effect-transistor measurements, we found that, while the Hall carrier types were always correct, the Hall carrier densities were overestimated by up to four orders of magnitude. This significant overestimation indicates that thin films of one-dimensional SWCNTs are quite different from conventional hopping transport systems.

## Introduction

Hall-effect measurements^1^ are one of the most direct and accurate methods for determining the type and density of charge carriers in crystalline materials. However, it is known that Hall measurements can lead to erroneous carrier types and densities when localized (or hopping) carriers, as opposed to delocalized (or band) carriers, dominantly contribute to the charge transport^2–13^. For example, in amorphous materials^9–12^ and organic polymers^13^, Hall measurements sometimes show a “sign anomaly,” i.e., a carrier type opposite to that determined through field-effect transistor (FET) and/or thermoelectric measurements. In addition, in some materials, even when the carrier type can be correctly determined, the carrier density cannot be properly evaluated by Hall measurements^2–8^. In systems where localized and delocalized carriers coexist, the Hall carrier density is overestimated because hopping carriers tend to reduce the Hall voltage^3^. Furthermore, in a system with grain boundaries, density overestimation occurs due to a reduction of the Hall electric field at grain boundaries^7^. Therefore, in disordered or heterogeneous materials, it is essential to compare Hall carrier types and density with those obtained by other methods such as FET measurements.

Macroscopic assemblies of single-wall carbon nanotubes (SWCNTs), such as FETs and transparent conducting films^14–16^, provide an intriguing system in which to study the Hall effect. SWCNT thin films are three-dimensional networks formed by one-dimensional nanoobjects (single ropes or bundles of SWCNTs), where carrier transport can be understood by a heterogeneous model of one-dimensional nanoobjects and junctions between them^17^. Since the Hall effect is usually generated by delocalized carriers, coherent conduction in one-dimensional nanoobjects, rather than hopping conduction at junctions, will dominantly contribute to the Hall effect. In this case, it is not self-evident how the transverse Hall effect appears in such a narrow conduction path. Moreover, as mentioned above, the presence of the hopping conduction reduces the Hall voltages, and thus the Hall voltages in SWCNT thin films must be carefully analyzed. Although Hall-effect studies on SWCNT films have been conducted^18,19^, these issues have never been addressed. The unreasonably large carrier density estimation reported there (~ 10^22^ cm^−3^) indicates the importance of considering these issues. Furthermore, despite the presence of various types of SWCNTs, such as metallic and semiconducting nanotubes, only mixed samples have been used in previous Hall-effect studies without controlling them. Since carrier transport can be strongly affected by the type of SWCNTs^20,21^, it is important to examine the effect of the electronic structure of SWCNTs on the Hall effect.

In this study, we evaluated the validity of the Hall effect in SWCNT films by systematically comparing obtained results of Hall effect with those of simultaneous FET measurements. To investigate the effect of the electronic structure of SWCNTs on the Hall effect, various SWCNT films with different electronic structures, such as metallic, semiconducting, and their mixture, were prepared. The carrier types and carrier density were systematically controlled by in the FET structure using ionic liquid, and then Hall-effect data were evaluated. We found that Hall-determined carrier types are always consistent with FET-determined carrier types, but Hall-determined carrier densities were significantly larger than FET-determined densities by three to four orders of magnitude. These results were found to be common to all SWCNT films studied, regardless of the electronic structures and the sizes of SWCNT films.

## Results

**Figure 1:**
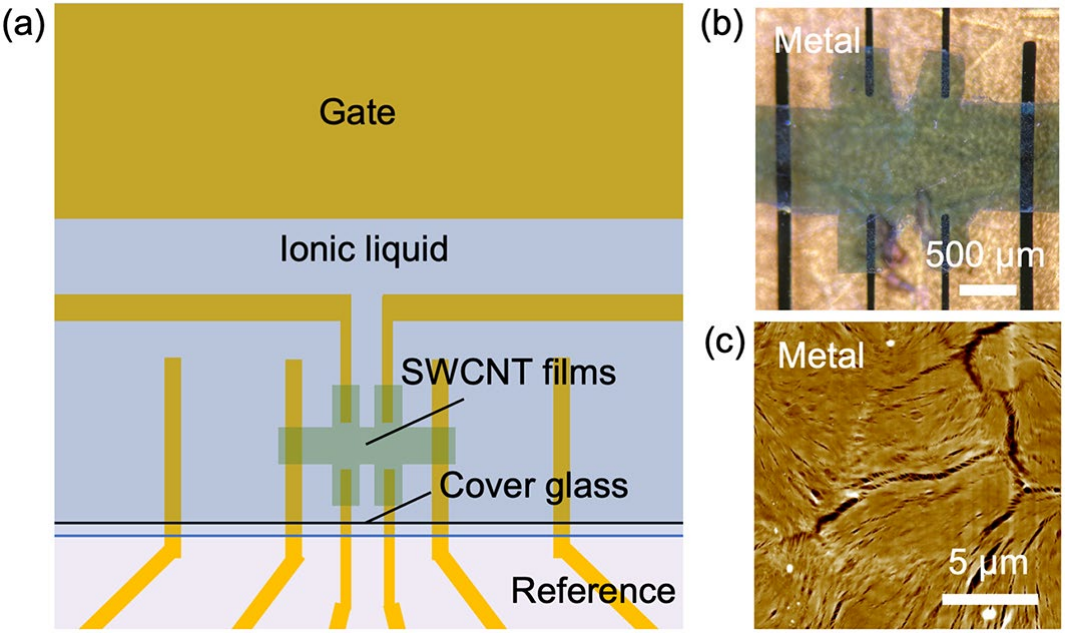
SWCNT thin film device. (**a**) Schematic of a SWCNT film device with standard Hall bar configuration. The yellow area represents Au (100 nm)/Ti (5 nm) films. The Au/Ti films have a higher sheet conductance than the gated SWCNT films, and only the Au/Ti films are considered as electrodes here. (**b**) Optical microscopy image of the SWCNT film device (Metal sample). (**c**) AFM (topography) image of the SWCNT film (Metal sample).

We fabricated FET devices with standard Hall bar configuration using SWCNT thin films, as shown in Fig. 1a and Fig. 1b (see also Supplementary Fig. S1). We prepared thin films of metallic (Metal), semiconducting (Semi), and unsorted SWCNTs (Mix) by filtration through polycarbonate membranes, as described in previous studies^22^. In addition, for unsorted SWCNTs, less-packed films (L-Mix) were prepared by filtration through nitrocellulose membranes in order to assess the effect of morphology. Each prepared SWCNT film was transferred onto a SiO_2_/Si substrate with Au/Ti electrodes. Figure 1c shows a typical atomic-force microscope image of one of the films. As seen in the image, the SWCNTs are randomly oriented.

**Figure 2:**
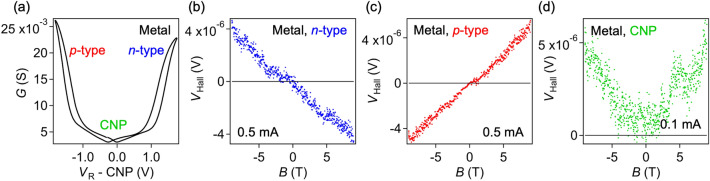
FET and Hall measurements of the SWCNT thin film devices. (**a**) Transfer characteristics of the Metal sample. Four-terminal conductance (*G*) is plotted as a function of reference voltage (*V*_R_). The data were measured at room temperature. (**b**–**d**) Hall voltage (*V*_Hall_) of the Metal sample, plotted as a function of magnetic field. Data at 200 K in the *n*-type region (*V*_R_ − CNP = 1.65 V) (**b**), in the *p*-type region (*V*_R_ − CNP = − 1.56 V) (**c**), and at the charge neutral point (CNP) (**d**) are shown.

Hall measurements were performed in vacuum. During the measurements, the carrier type and density were controlled by varying the gate voltage. Figure 2a shows typical transfer characteristics of the Metal device, plotted as a function of reference voltage, *V*_R_ (see Supplementary Fig. S2 for other devices). All devices exhibit ambipolar transistor behavior (Supplementary Fig. S2), which shows *p*-type and *n*-type current amplification with the charge neutral point (CNP) in the middle. The on/off ratio reached ~ 10 for the Metal, Mix, and L-Mix samples and ~ 10^4^ for the Semi samples, indicating that carrier doping is effectively achieved by the ionic-liquid gating method. We measured the Hall voltage across the channel under a constant current while sweeping the magnetic field. To avoid temporal changes in the carrier density during the measurements, all measurements were performed at temperatures below the freezing point of the ionic liquid (~ 256 K)^23^.

Figure 2b,c shows the measured Hall voltage of the Metal sample in the *n*-type (*p*-type) region as a function of magnetic field (see Supplementary Fig. S3 for other data plotted as a function of time). The sign of the Hall voltage changes when the direction of the magnetic field is reversed, and the sign depends on the carrier type. When holes (electrons) are the dominant carriers, the Hall voltage is positive (negative) at positive magnetic fields, as expected. This behavior was observed in relatively high-density regions, and was similarly observed in different temperature regions above 50 K (Supplementary Fig. S4). However, in low-density regions (Fig. 2d) and low-temperature regions below 30 K (Supplementary Fig. S4), it was difficult to observe this ideal reversal response of the Hall voltage. In these regions, the transverse Hall voltage could not be precisely evaluated because of the large longitudinal resistance and the presence of magnetoresistance, as discussed below. Therefore, in this study, we only discuss the Hall voltage in the high carrier density region.

**Figure 3:**
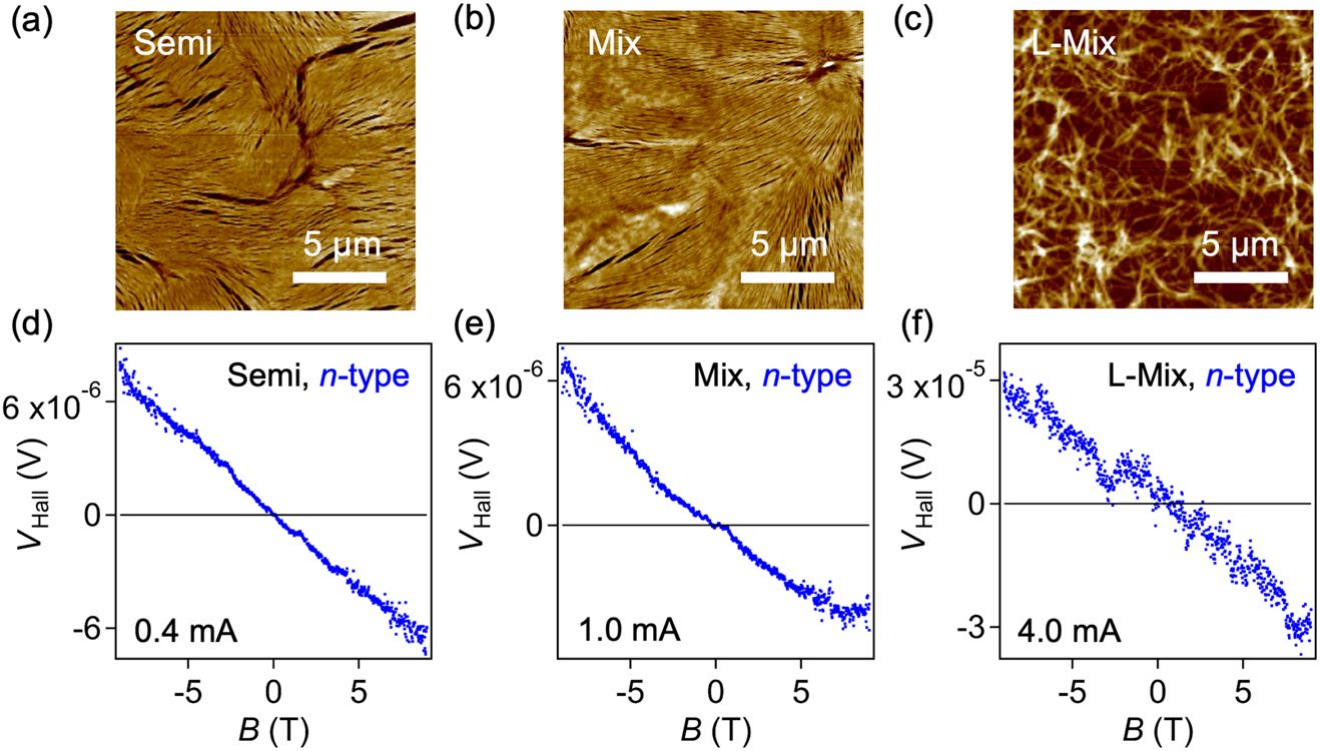
Hall measurements of various SWCNT thin film devices. (**a**–**c**) AFM images of the Semi (**a**), Mix (**b**), and L-Mix samples (**c**). (**d**–**f**) Hall voltage (*V*_Hall_) of the devices shown in (**a**–**c**) in the *n*-type region as a function of magnetic field. Data at 200 K for the Semi (*V*_R_ − CNP = 1.60 V) (**d**), Mix (*V*_R_ − CNP = 1.43 V) (**e**), and L-Mix samples (*V*_R_ − CNP = 1.39 V) (**f**) are shown.

Figure 3 shows Hall-measurement results for the Semi, Mix, and L-Mix samples in the high-density *n*-type region; see Supplementary Fig. S5 for results in the *p*-type region. In all cases, the sign of the Hall voltage was consistent with that obtained in FET measurements. This “sign consistency” was universally confirmed in all SWCNT films studied, regardless of their electronic structures. However, it was more difficult to obtain the ideal reversal response of the Hall voltage in the Mix and L-Mix samples than in the Metal and Semi samples. The ideal responses in the Mix and L-Mix samples were only observed at temperatures above 150 K (see Supplementary Fig. S6). This emphasizes the important fact that the coexistence of different electronic structures can negatively affect Hall-effect results in SWCNT films.

Next, we investigated whether the carrier density could be properly evaluated by Hall measurements. The Hall carrier density *n*_Hall_ was derived from the corrected Hall voltage *V*_Hall_ using

1 $${n}_{\text{Hall}}=\frac{1}{e}\times \frac{I\cdot B}{{V}_{\text{Hall}}}$$

where *I* is the source-drain current and *B* is the magnetic field applied perpendicular to the film. The estimated Hall carrier density was compared to the FET carrier density *n*_FET_, which was derived from the FET specific capacitance *c*_FET_ (see Supplementary Fig. S7) using

2 $${n}_{\text{FET}}=\frac{1}{e}\int {c}_{\text{FET}}d{V}_{\text{R}}$$

**Figure 4:**
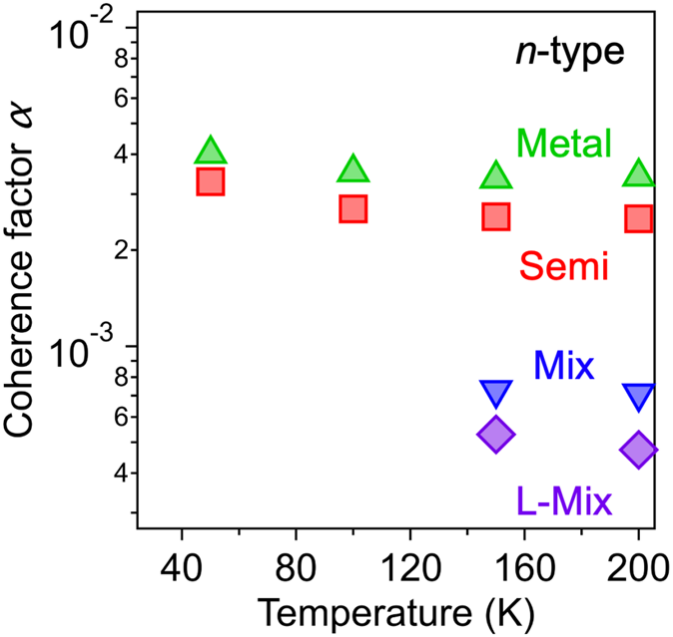
Coherence factor (*α*) of the various SWCNT thin film devices in the *n*-type region as a function of temperature. Data for the Metal (green), Semi (red), Mix (blue), and L-Mix samples (purple) are shown.

The measured *c*_FET_ shows different values depending on the measurement frequency. In this study, we employed the *c*_FET_ at 200 mHz, which was confirmed to be low enough frequency for the formation of the electric double layer and appropriate for FET measurements (see Supplementary Fig. S8). Table 1 shows a summary of the Hall and FET carrier densities of all the samples obtained in the *n*-type region (see Supplementary Table S1 for results in the *p*-type region). We found that in all devices the Hall carrier density (~ 10^17^ cm^−2^) was more than two orders of magnitude higher than the FET carrier density (~ 10^15^ cm^−2^). Judging from the capacitance measurement, the *n*_FET_ values determined from the capacitance at 200 mHz are reasonable, and even if there is slight change in the capacitance due to the difference of measurement time scale, it is negligible compared to the difference of several orders of magnitude in the two carrier densities (see Supporting information for details). Therefore, *n*_Hall_ values are overestimated. Regarding the three-dimensional *n*_Hall_, we assume that the entire SWCNT films (59–93 nm in thickness) are doped, since previous studies have shown that when SWCNT films are gated through ionic liquid, changes in optical properties due to the Fermi level shift occur in the whole of the SWCNT film^24,25^. The obtained three-dimensional *n*_Hall_ values correspond to ~ 10^22^ cm^−3^, reproducing the extremely large carrier density reported in the previous study^19^. On the other hand, the obtained *n*_FET_ values correspond to ~ 10^20^ cm^−3^, which is comparable to those typically attained by electrolyte gating^26^. The values of the so-called coherence factor *α*, which is defined as the ratio of the two-dimensional carrier densities, ${n}_{\text{FET}}/{n}_{\text{Hall}}$, are also summarized in Table 1. The obtained values of *α* (~ 10^–3^) are much smaller than those reported for organic semiconductors and polymer materials (> 0.1)^2–6,27^. Figure 4 shows the temperature dependence of *α* (see also Supplementary Fig. S9), exhibiting a slight monotonic increase with decreasing temperature, similar to organic semiconductors^4^, although the absolute values are very different. To check the reproducibility of these extremely small *α* values, we performed additional measurements on devices with different channel sizes, and the results were fully reproduced (see Supplementary Fig. S10). These results suggest that the small *α* values are not influenced by the device structure.

**Table 1:** FET carrier density (*n*_FET_), Hall carrier density (*n*_Hall_), coherence factor (*α*), FET carrier mobility (*μ*_FET_) of the various SWCNT thin film devices studied in this work.

Samples	*n*_FET_ (cm^−2^)	*n*_Hall_ (cm^−2^)	*α*	*μ*_FET_ (cm^2^ V^−1^ s^−1^)
Metal	2.4 × 10^15^	7.0 × 10^17^	3.4 × 10^–3^	52
Semi	7.8 × 10^14^	3.1 × 10^17^	2.5 × 10^–3^	62
Mix	7.9 × 10^14^	1.1 × 10^18^	7.1 × 10^–4^	1.0 × 10^2^
L-Mix	3.5 × 10^14^	7.5 × 10^17^	4.7 × 10^–4^	75

Data taken at 200 K in the *n*-type region are shown.

## Discussion

We reiterate that we did not observe any Hall-coefficient sign anomaly in any of our films, although such a phenomenon is often observed in hopping-conduction-dominated systems^28–31^. The observed sign consistency indicates the existence of delocalized carriers that respond to the Lorentz force, as opposed to the hopping carriers whose existence is clear from the temperature dependence of resistance (see Supplementary Fig. S11)^21,28,31^. The existence of weakly localized carriers was confirmed by the appearance of negative magnetoresistance in any conditions (Supplementary Figs. S12, 13), as described as a signature of weak localization^20,21^. Therefore, we assume that such weakly localized carriers contributed the Hall voltage and ensured the sign consistency in the Hall effect in our SWCNT films.

Next, we discuss the possible origins of the very small *α* values we observed. First, the Hall voltage can be compensated by the hopping carriers moving in the direction opposite to the Lorentz force^3^. In organic semiconductors, this contribution is usually related to the reduction of carrier mobility. Table 1 shows the four-terminal FET carrier mobility (${\mu }_{\text{FET}}$) values for the SWCNT film devices, calculated using the formula

3 $${\mu }_{\text{FET}}=\frac{L}{{Wc}_{\text{FET}}}\times \frac{dG}{{dV}_{\text{R}}}$$

where *L* is the channel length, *W* is the channel width, and *G* is the four-terminal conductance. We note that our SWCNT films exhibited relatively high carrier mobilities (> 52 cm^2^ V^−1^ s^−1^), comparable to those reported for similar gated SWCNT films (~ 59 cm^2^ V^−1^ s^−1^)^32,33^. However, these mobilities are much smaller than that expected for a single SWCNT rope (> 1,000 cm^2^ V^−1^ s^−1^)^34^, indicating the contribution of hopping conduction that can reduce *α*. The effect of grain boundaries, reported in organic composite systems, can be also the origin to reduce the Hall voltage^7^. SWCNT films have many interfaces, both tube-tube and bundle-bundle junctions (see AFM images in Figs. 1 and 3), which can be thought of as grain boundaries in the context of carrier localization. However, the above two contributions are generally used to explain the decrease in *α* from 1, which represents the ideal Hall effect, to about 0.1, and this extremely small *α* (~ 10^–4^) obtained for the SWCNT films suggests that there may be other factors in addition to the above two. We assume the following effect of dimensionality. If the conduction path (e.g., a SWCNT bundle) of the delocalized carriers is closer to one-dimensional, their contribution to the two-dimensional Hall effect should be smaller, resulting in smaller *α*.

Finally, we highlight two trends of *α* values observed in sample dependence: (i) *α* (Metal) > *α* (Semi) > *α* (Mix), and (ii) *α* (Mix) > *α* (L-Mix). These trends suggest that the aforementioned factors that affect the Hall voltage, such as weak localization, hopping conduction, grain boundaries, depend on the sample. The Metal sample exhibited the largest *α*, which is reasonable because it shows weak localization even without carrier doping^20,21^. The Mix samples containing metallic SWCNTs showing a smaller *α* than the Semi sample indicates that *α* in the high-density region is affected not only by the electronic structure but also by sample inhomogeneity. Regarding (ii), it is noteworthy that there is no significant difference in *α* between the Mix and L-Mix samples, despite the significant difference in film morphology (see Fig. 3). This suggests that the morphology of films does not considerably affect the Hall effect.

In conclusion, we evaluated the validity of the Hall effect in SWCNT films with various electronic structures and morphologies. We observed clear Hall voltages in all samples in high carrier density regions and confirmed that the carrier types were consistent between the Hall effect and FET measurements. However, the Hall carrier densities were significantly overestimated, compared to the FET densities, resulting in extremely small values of *α* (< 10^–3^). The values of *α* for SWCNT films are the smallest among the values reported for all the materials, indicating that thin films of one-dimensional SWCNTs are quite different from conventional hopping transport systems. This study suggests that, even though Hall measurements are a general method to evaluate carrier types and carrier densities, a careful evaluation is crucial for correct estimation of the carrier density when interpreting Hall-effect results for SWCNT films.

## Methods

### Preparation of SWCNT films

Metallic and semiconducting SWCNTs were separated from ArcSO SWCNTs (1.4 nm in diameter, Meijo Nano Carbon Co.) by density gradient ultracentrifugation^20^. After separation, the SWCNT dispersion was treated by ultrafiltration (Amicon Ultra-15 with Ultracel-100 membrane, Merck Millipore) to remove density gradient medium and disperse the SWCNTs in an aqueous solution of 0.3% sodium deoxycholate (DOC). The dispersion was sonicated with a tip sonicator (20% in power, Sonifier 250D, Branson) for 30 min and ultracentrifuged for 1 h at 36,000 rpm using a swing bucket rotor (P40ST, Hitachi Koki). The top 80% of the supernatant was collected. Random oriented films were prepared by filtration through polycarbonate (110605, 0.1 μm pore size, Whatman) or nitrocellulose membranes (GSWP, 0.22 μm pore size, Merck Millipore).

### Device fabrication

The obtained SWCNT film was transferred onto a SiO_2_/Si substrate with Au/Ti electrodes by removing the membrane using chloroform and acetone. The film was annealed at 200 °C for 2 h in a vacuum to remove residual organic solvent. Ionic liquid N,N,N-trimethyl-N-propylammonium bis(trifluoromethanesulfonyl)-imide (TMPA-TFSI, Kanto Chemical Co., Inc.) was dropped onto the SWCNT device and held on the device by putting the cover glass on top.

### FET measurements

FET measurements were performed in vacuum inside the physical properties measurement system (PPMS, Quantum Design Co.). The experimental setup is shown in Supplementary Fig. S14. A source meter (2636A, Keithley) was used to apply the gate voltage and drain current to the device. To evaluate the actual voltage applied to the device, the reference voltage *V*_R_ was measured with a digital multimeter (2000, Keithley). The four-terminal conductance was measured with a digital multimeter (2000, Keithley) to exclude the effect of contact resistance.

### Specific capacitance measurements

Specific capacitance of the SWCNT film device was measured at 200 mHz with an impedance analyzer (611ES, ALS Co. Ltd.). This method allows us to measure the total capacitance (${C}_{\text{total}}$) of the series-connected electric double layer capacitors at the gate electrode (${C}_{\text{gate}}$) and at the SWCNT film (${C}_{\text{SWCNT}}$), where ${C}_{\text{SWCNT}}$ consists of the geometrical capacitance of SWCNT film (${C}_{\text{G}}$) and quantum capacitance (${C}_{\text{Q}}$) of SWCNTs. Following the literature^26^, these relations are represented as $1/{C}_{\text{total}}=1/{C}_{\text{SWCNT}}+1/{C}_{\text{gate}}$ and $1/{C}_{\text{SWCNT}}=1/{C}_{\text{G}}+1/{C}_{\text{Q}}$. ${C}_{\text{SWCNT}}$ were calculated from the measured ${C}_{\text{total}}$ using the well-known voltage-ratio (area-ratio) relationship for series circuits of capacitors^32^. The ${\Delta V}_{\text{G}}$ and ${\Delta V}_{\text{R}}$, which are applied to ${C}_{\text{total}}$ and ${C}_{\text{SWCNT}}$, respectively, were measured. Finally, the specific capacitance (${c}_{\text{FET}}$) of the SWCNT sample was derived using ${c}_{\text{FET}}={C}_{\text{SWCNT}}/{A}_{\text{SWCNT}}$, where ${A}_{\text{SWCNT}}$ is the area of SWCNT film.

### Magnetoresistance and Hall effect measurements

To avoid temporal changes in carrier density during the measurements, Magnetoresistance and Hall effect measurements were performed at temperatures below the freezing point of the ionic liquid (~ 256 K), below which the carrier density is fixed without the gate voltage. The gate voltage was applied to the device until the gate current settled down, and the device was subsequently cooled down to a temperature below the freezing point with the gate voltage applied. For the reference voltage, we used that in the FET measurements, referring to the four-terminal conductivity at room temperature. For magnetoresistance measurements, the four-terminal longitudinal resistance was measured under a constant current while sweeping the magnetic field between 9 T and − 9 T. For Hall effect measurements, the transverse Hall voltage was measured using nanovoltmeter (181, Keithley). The offset voltage at zero field was reduced by a potentiometer. To accurately calculate the Hall carrier density, we used the Hall voltage ${V}_{\text{Hall}}$ corrected by conventional method, ${V}_{\text{Hall}}=\left(1/2\right)\left[V\left(B\right)-V\left(-B\right)\right]$, where *V* is the raw data of Hall voltage and *B* is the magnetic field.

## Supplementary Information

Supplementary Information.
