# Supplementary material for: Hall effect in gated single-wall carbon nanotube films
Source: Sci Rep. 2022 Jan 7;12:101. doi: 10.1038/s41598-021-03911-7 (PMC8741975; doi:10.1038/s41598-021-03911-7)
Supplement: Supplementary file 1 — Supplementary Information. [file 41598_2021_3911_MOESM1_ESM.pdf]

Supplementary Information for

Hall effect in gated single-wall carbon nanotube films

Yohei Yomogida,*[1] Kanako Horiuchi,[1] Ryotaro Okada,[1] Hideki Kawai,[1] Yota Ichinose,[1] Hiroyuki Nishidome,[1] Kan Ueji,[1] Natsumi Komatsu,[2] Weilu Gao,[3] Junichiro Kono,[2,4,5] and Kazuhiro Yanagi*[1]

[1]Department of Physics, Tokyo Metropolitan University, Hachioji, Tokyo 192-0397, Japan

[2]Department of Electrical and Computer Engineering, Rice University, Houston, Texas 77005, USA

[3]Department of Electrical and Computer Engineering, University of Utah, Salt Lake City, UT 84112, USA

[4]Department of Physics and Astronomy, Rice University, Houston, Texas 77005, USA

[5]Department of Materials Science and NanoEngineering, Rice University, Houston, Texas 77005, USA

E-mail: yomogida@tmu.ac.jp, yanagi-kazuhiro@tmu.ac.jp

The details of FET carrier density

The FET carrier density (n_{FET}) is derived from the specific capacitance (c_{FET}), which is obtained by dividing the measured capacitance by the area or volume of the SWCNT film. Supplementary Fig. S8 shows the frequency dependence of the c_{FET}. The formation of the electric double layer is observed below 1 Hz, and we employed c_{FET} at 200 mHz, which was low enough frequency for the formation of the electric double layer and appropriate for FET measurements. On the other hand, there are slow response components, which cause a slight change in c_{FET} between 1 Hz and 10 mHz. The duration time at each gate voltage is different between the FET and Hall measurements. In the FET measurements, the duration time scale is 10 s, and in the Hall measurements, the time scale is 10 min. Therefore, due to the difference in the measurement time scale between the FET and Hall measurements, the capacitance may be slightly different. However, considering the slight change in c_{FET} (42 to 93 μF cm^{-2}) over a wide frequency

range (1 Hz to 10 mHz), the difference in n_{FET} is negligible compared to the difference of several orders of magnitude in the two carrier densities, and does not affect the significantly small α values."

Figures and Tables

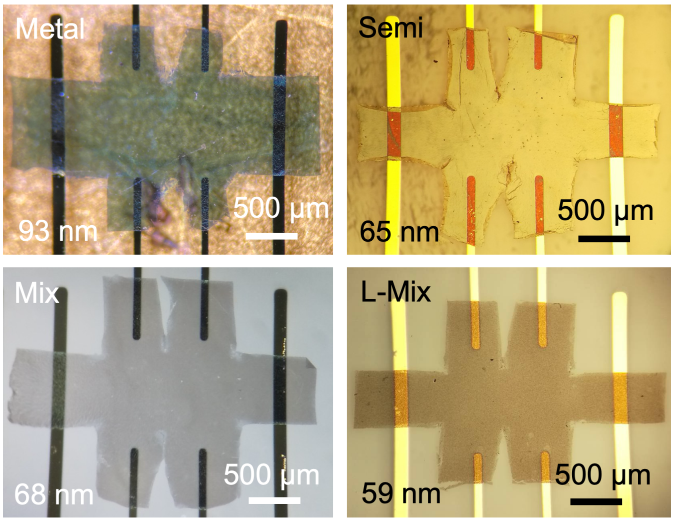

Fig. S1. Optical microscopy images of the various SWCNT thin film devices. The film thickness is also shown.

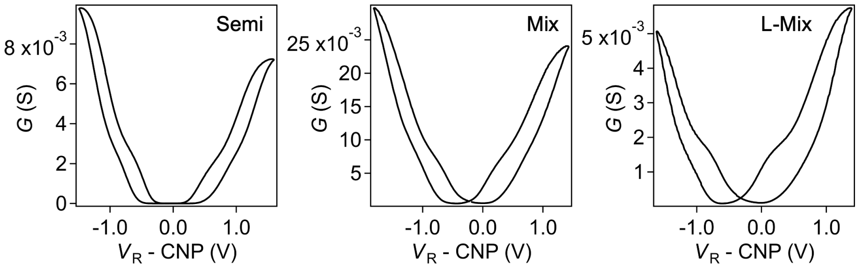

Fig. S2. Transfer characteristics of the various SWCNT thin film devices. Four-terminal conductance (G) is plotted as a function of the reference voltage (V_{R}). Data for the Semi (left), Mix (center), L-Mix (right) samples are shown. The data were measured at room temperature.

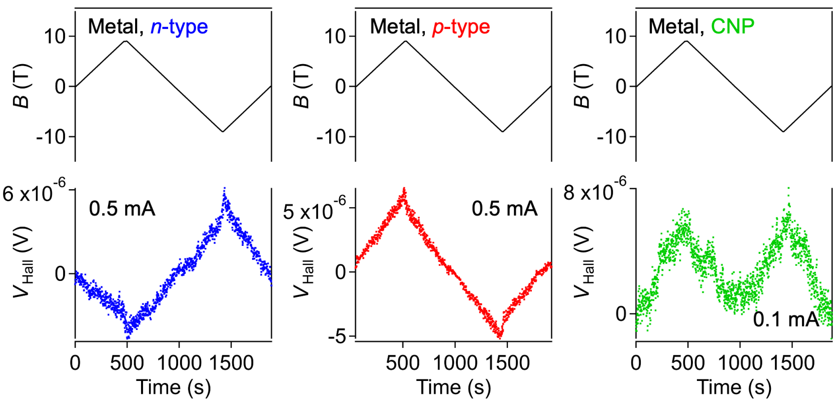

Fig. S3. Hall voltage (V_{Hall}) of the Metal sample, plotted as a function of the time. Data at 200 K in the n-type region (V_{R} − CNP = 1.65 V) (left), in the p-type region (V_{R} − CNP = −1.56 V) (center), and at the charge neutral point (CNP) (right) are shown along with the magnetic field profile (black line).

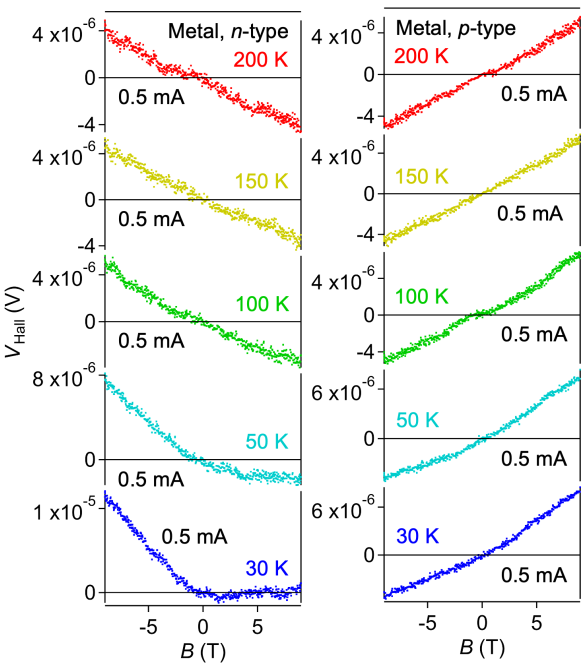

Fig. S4. Hall voltage (V_{Hall}) at different temperatures for the Metal sample, plotted as a function of the magnetic field (B). Data in the n-type (V_{R} − CNP = 1.65 V) (left) and p-type regions (V_{R} − CNP = −1.56 V) (right) are shown.

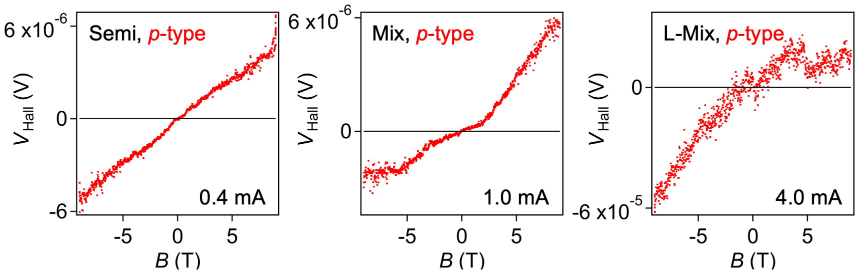

Fig. S5. Hall voltage (V_{Hall}) of the various SWCNT thin film devices in the p-type region, plotted as a function of the magnetic field (B). Data at 200 K for the Semi ($V_{\mathrm{R}} - \mathrm{CNP} = -1.33$ V) (left), Mix ($V_{\mathrm{R}} - \mathrm{CNP} = -1.35$ V) (center), L-Mix samples ($V_{\mathrm{R}} - \mathrm{CNP} = -0.99$ V) (right) are shown.

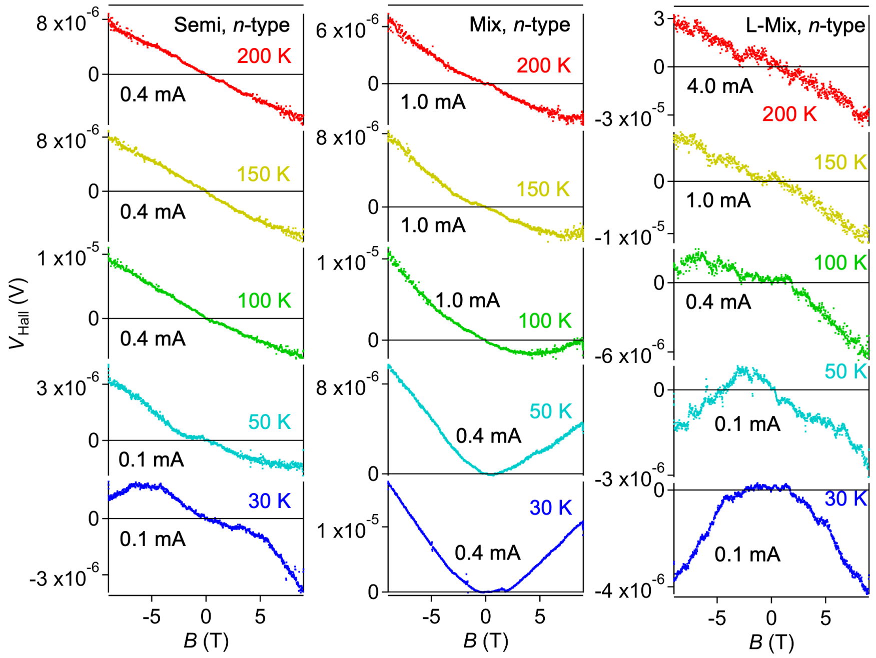

Fig. S6. Hall voltage (V_{Hall}) at different temperatures for the various SWCNT thin film devices, plotted as a function of the magnetic field (B). Data in the n-type region for the Semi ($V_{\mathrm{R}} - \mathrm{CNP} = 1.60$ V) (left), Mix ($V_{\mathrm{R}} - \mathrm{CNP} = 1.43$ V) (center), and L-Mix samples ($V_{\mathrm{R}} - \mathrm{CNP} = 1.39$ V) (right) are shown.

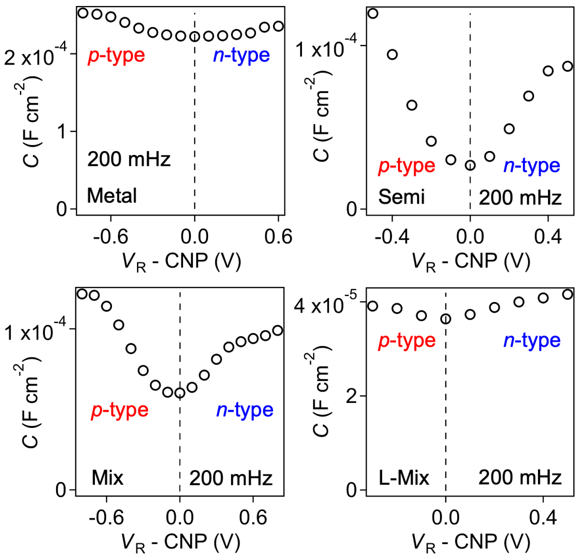

Fig. S7. Specific capacitance (c_{FET}) of the various SWCNT thin film devices, plotted as a function of the reference voltage (V_R). Data for the Metal (top, left), Semi (top, right), Mix (bottom, left), L-Mix samples (bottom, right) are shown. The frequency in the measurements is also shown.

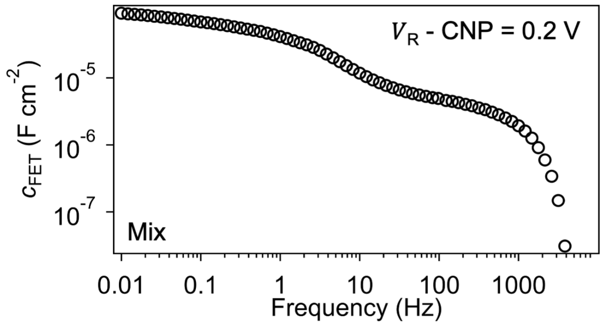

Fig. S8. Specific capacitance (c_{FET}) of the Mix sample, plotted as a function of the frequency. The reference voltage in the measurement is also shown.

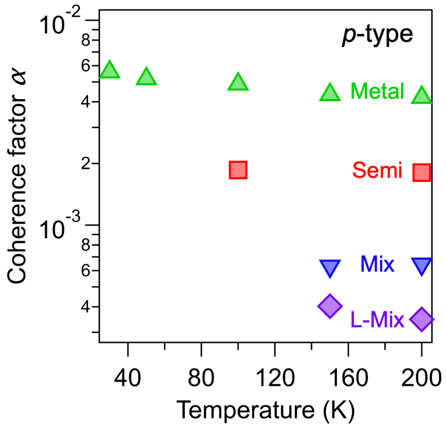

Fig. S9. Coherence factor (α) of the various SWCNT thin film devices in the p-type region, plotted as a function of the temperature. Data for the Metal (green), Semi (red), Mix (blue), and L-Mix samples (purple) are shown.

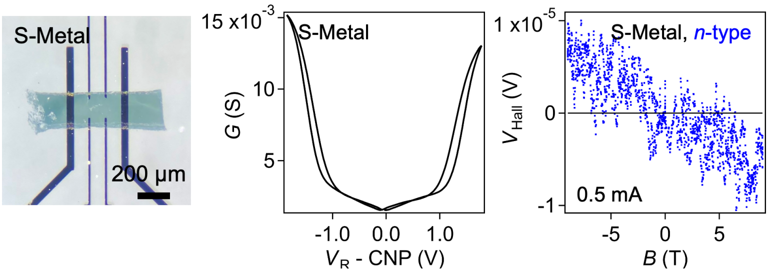

Fig. S10. FET and Hall measurements of the Metal sample with small size (S-Metal). Optical microscopy images of the S-Metal sample (left). Transfer characteristics of the S-Metal sample (center). Four-terminal conductance (G) is plotted as a function of the reference voltage (V_R). Hall voltage (V_{Hall}) of the S-Metal sample (right), plotted as a function of the magnetic field (B). Data at 50 K in the n-type region ($V_R - CNP = 1.52$ V) are shown. The coherence factor is estimated to be 4.5×10^{-3}, which is comparable to the value of 4.0×10^{-3} at 50 K for the Metal sample.

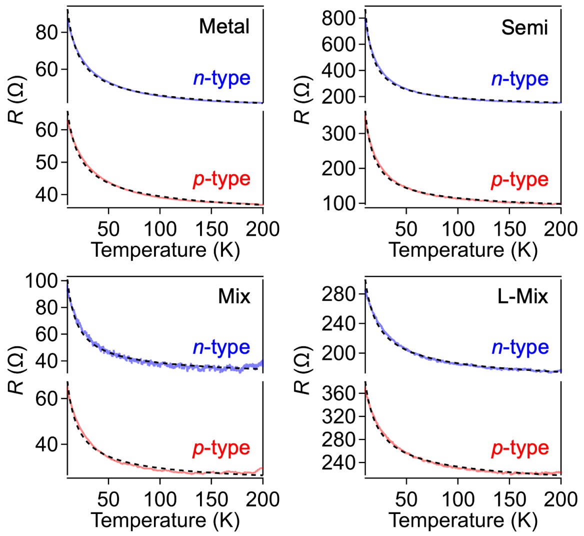

Fig. S11. Temperature dependence of four-terminal resistance (R) of the various SWCNT thin film devices. Data in the n-type (blue) and p-type regions (red) for the Metal ($V_R - \mathrm{CNP} = 1.65$ V, -1.56 V) (top, left), Semi ($V_R - \mathrm{CNP} = 1.60$ V, -1.33 V) (top, right), Mix ($V_R - \mathrm{CNP} = 1.43$ V, -1.35 V) (bottom, left), and L-Mix samples ($V_R - \mathrm{CNP} = 1.39$ V, -0.99 V) (bottom, right) are shown. All data were analyzed using the following variable range hopping (VRH) model, $R = R_0 \exp\left[(T_0/T)^{1/(d+1)}\right]$, where d is 1, 2, or 3. All data were in some agreement with the VRH model for $d = 2$ or $d = 3$. The dotted lines show the data for $d = 2$.

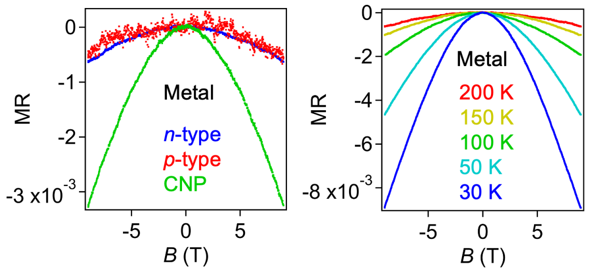

Fig. S12. Magnetoresistance ($(R(B) - R(0))/R(0)$) of the Metal sample in different carrier density regions (left) and different temperature regions (right). (left) Data at 200 K in the n-type region ($V_R - \mathrm{CNP} = 1.65$ V) (blue), in the p-type region ($V_R - \mathrm{CNP} = -1.56$ V) (red), and at the charge neutral point (CNP) (green) are shown. (right) Data in the n-type region ($V_R - \mathrm{CNP} = 1.65$ V) are shown. All data show the negative magnetoresistance.

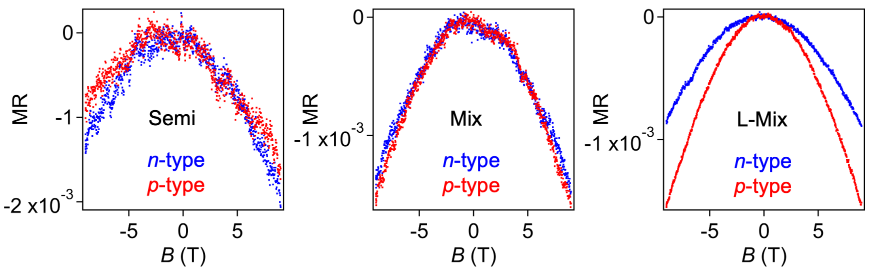

Fig. S13. Magnetoresistance $((R(B) - R(0))/R(0))$ of the various SWCNT thin film devices in the n-type (blue line) and p-type regions (red line). Data at 200 K for the Semi (V_R − CNP = 1.60 V, −1.33 V) (left), Mix (V_R − CNP = 1.43 V, −1.35 V) (center), and L-Mix samples (V_R − CNP = 1.39 V, −0.99 V) (right) are shown. All data show the negative magnetoresistance.

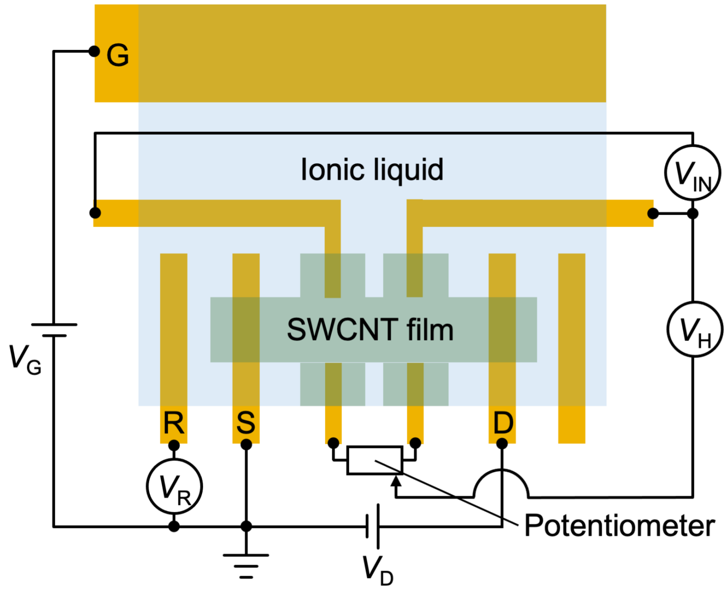

Fig. S14. Schematic of the experimental setup for FET and Hall measurements. The Au/Ti electrodes labeled S, D, G, and R, represent the source, drain, gate, and reference electrodes, respectively. A source meter was used to apply the gate (V_G) and drain voltage (V_D) to the device. Digital multimeters (V_R and V_{IN}) were used to measure the reference voltage and four-terminal conductance. For the Hall measurements, in addition to the above, a nanovoltmeter (V_H) was used to measure the Hall voltage. A potentiometer was used to remove the offset voltage at zero field.

Table S1. Hall carrier density (n_{Hall}), FET carrier density (n_{FET}), and coherence factor (α) of the various SWCNT thin film devices. Data at 200 K are shown.

Types	Samples	V_R – CNP (V)	n_{FET} (cm^{-2})	n_{Hall} (cm^{-2})	α
n-type	Metal	1.68	2.4×10^{15}	7.0×10^{17}	3.4×10^{-3}
	Semi	1.60	7.8×10^{14}	3.1×10^{17}	2.5×10^{-3}
	Mix	1.43	7.9×10^{14}	1.1×10^{18}	7.1×10^{-4}
	L-Mix	1.39	3.5×10^{14}	7.5×10^{17}	4.7×10^{-4}
p-type	Metal	–1.56	2.3×10^{15}	5.6×10^{17}	4.2×10^{-3}
	Semi	–1.33	7.8×10^{14}	4.3×10^{17}	1.8×10^{-3}
	Mix	–1.35	9.0×10^{14}	1.4×10^{18}	6.4×10^{-4}
	L-Mix	–0.99	2.4×10^{14}	6.9×10^{17}	3.4×10^{-4}
